# Unraveling progressive verbal memory deficits in Huntington’s disease: insights from the LASSI-L

**DOI:** 10.3389/fneur.2025.1494816

**Published:** 2025-02-19

**Authors:** Luis A. Sierra, Japleen Kaur, Samuel A. Frank, Mark A. Halko, Rosie E. Curiel Cid, David A. Loewenstein, Jody Corey-Bloom, Simon Laganiere

**Affiliations:** ^1^Department of Neurology, Beth Israel Deaconess Medical Center, Boston, MA, United States; ^2^Department of Neurosciences, School of Medicine, University of California, San Diego, San Diego, CA, United States; ^3^Harvard Medical School, Boston, MA, United States; ^4^Department of Psychiatry, McLean Hospital, Belmont, MA, United States; ^5^Center for Cognitive Neuroscience and Aging and Department of Psychiatry and Behavioral Sciences, Miller School of Medicine, University of Miami, Miami, FL, United States

**Keywords:** semantic interference, cognition, Huntington’s disease, executive function, premotor manifest HD, LASSI-L

## Abstract

**Background and objectives:**

Huntington’s disease (HD) is characterized by progressive cognitive decline, with early deficits often preceding motor symptoms. The Loewenstein-Acevedo Scales for Semantic Interference and Learning (LASSI-L) captures many types of deficits in verbal memory including susceptibility to interference. The current study aims to delineate the progression of these deficits across different stages of HD.

**Methods:**

151 participants (89 HD, 62 healthy controls) were recruited across three sites. HD participants were classified into three groups using a PIN score and TMS: >10 years from manifest HD, <10 years from manifest HD, and manifest HD. Group comparisons on the LASSI-L were assessed using multiple ANCOVAs adjusting for age, followed by post-hoc pairwise comparisons and a Bonferroni correction.

**Results:**

Sequential group differences for susceptibility to interference effects were observed on the LASSI-L. Proactive Semantic Interference (PSI) deficits emerged >10 years before manifest HD, Failure to Recover from PSI (*fr*PSI) emerged <10 years before manifest HD, and in the Manifest HD stage, participants exhibited additional deficits in Retroactive Semantic Interference (RSI). Based on cutoff scores derived from healthy control performance, 98% (87/89) of the HD cohort demonstrated either normal performance or significant impairments, primarily in PSI, with some showing concurrent deficits in *fr*PSI and/or RSI. Furthermore, 88% of participants adhered to the full sequential decline pattern, progressing from deficits in PSI, to *fr*PSI, and then to RSI.

**Discussion:**

The LASSI-L appears to be a useful tool for detecting early and progressive cognitive changes in Huntington’s disease, particularly by capturing the sequential nature of verbal memory deficits, including early vulnerability to interference. These findings suggest that the LASSI-L may help refine HD staging by integrating sequential neuropsychological markers of cognitive decline.

## Introduction

Huntington’s disease (HD) is a hereditary neurodegenerative disorder characterized by progressive impairment in motor function, cognition, and behavior. The diagnosis of motor manifest HD has traditionally relied on identifying distinct motor symptoms; however, cognitive decline often precedes these indicators (1–5). This observation underscores the need for precise and sensitive cognitive assessments that can identify early markers of decline and track disease progression over time, helping to improve the accuracy of disease staging systems (6).

Research consistently identifies executive dysfunction and processing speed as the earliest cognitive abilities to decline in HD (7–9). Although patients and their families report memory impairment as significantly impactful (10), these deficits are often not detected in the early stages of the disease. Traditional testing paradigms, such as object recall and the Hopkins Verbal Learning Test-Revised (HVLT-R), generally identify memory deficits only in the later stages of HD (11, 12).

However, certain memory task paradigms are more sensitive to the effects of executive function and processing speed than others (13). This manifests, for example, in measures such as susceptibility to interference, intrusion errors, and variability in the speed and efficiency of encoding and retrieval (14–17). As a result, specific aspects of memory—particularly those heavily dependent on executive function and processing speed —may be inadequately challenged by traditional testing paradigms. In support of this claim, Sierra et al. (4) found that individuals with Huntington’s disease (HD) exhibit heightened susceptibility to proactive interference at early stages, years before the manifestation of motor symptoms. These subtle and progressive deficits likely become apparent at different stages of HD, with susceptibility to proactive interference potentially preceding other measures such as retroactive interference and/or intrusion errors.

A key priority for the HD research community is the accurate staging of the disease, which requires a detailed understanding of how various traits, including all aspects of cognitive performance, deteriorate over time. A more precise understanding of the progression of these verbal memory deficits could significantly improve the categorization of HD stages across populations and studies, thereby enhancing and refining classification systems such as the Huntington’s Disease Integrated Staging System (HD-ISS) (18). However, traditional verbal memory tests have struggled to consistently capture the precise sequence of memory decline in HD, particularly in relation to proactive and retroactive interference and intrusion errors, highlighting the utility of additional diagnostic tools.

To address this gap, our study employed the Loewenstein-Acevedo Scales for Semantic Interference and Learning (LASSI-L) to assess verbal memory in a large cohort of Huntington’s Disease (HD) patients across various stages of disease progression, from pre-motor manifest to advanced stages. The LASSI-L is designed to assess various aspects of memory, challenging executive function, processing speed, and memory retrieval simultaneously. This semantic interference paradigm significantly influences memory processes and is particularly effective in revealing short-term memory deficits (19).

The LASSI-L is a verbal learning test combining semantic cueing with alternating free and cued recall of salient semantic categories to optimize encoding and induce heightened interference effects (19, 20). It uses two semantically related 15-word lists (A and B) from three semantic categories (fruits, clothing, musical instruments). Participants read each word aloud, followed by free recall and category-cued recall tasks, while recall accuracy is recorded. This process measures the susceptibility to proactive semantic interference (PSI) through List A’s impact on List B recall and retroactive semantic interference (RSI) by List B’s impact on List A recall. Errors (intrusions) are noted throughout. Studies by Sierra et al. (4, 21) found significant proactive interference (PSI) in premanifest HD participants, who recovered well on subsequent attempts without showing susceptibility to retroactive interference (RSI).

The findings from the current study have the potential to enhance our understanding of how specific verbal memory deficits progress in HD over time. This improved understanding may contribute to more accurate staging and monitoring of disease progression. Additionally, the results could inform the development of verbal memory tests better suited to each stage of the disease, ultimately supporting more effective clinical management and targeted patient care (22).

## Methods

### Participants

Participants were recruited from Beth Israel Deaconess Medical Center in Boston, Massachusetts; the Huntington’s Disease Clinical Research Center at the University of California, San Diego (UCSD); and the Center for Cognitive Neuroscience and Aging at the University of Miami (UM). Of the 151 participants (89 HD and 62 HC), BIDMC enrolled 38 HD and 25 HC; UCSD enrolled 51 HD and 17 HC; and UM enrolled 20 HC. Healthy controls and individuals with Huntington’s Disease (HD), confirmed by genetic testing with a pathological expansion of mHTT (CAG ≥ 36), were enrolled. The study protocol received approval from BIDMC’s institutional review board, and all participants provided written consent. At UCSD, participants had previously completed the LASSI-L as part of a local registry assessment. At the University of Miami, control participants had completed the LASSI-L as part of the Assessment of Middle-aged Offspring of Late Alzheimer’s Probands (OLOAD) study. Additional inclusion criteria comprised an age range of 18–65. To reduce confounding effects, healthy control subjects were matched by sex, and education level to the preHD group. Exclusion criteria for both the HD and HC cohorts included:

- Any prior history of neurological disorders, such as stroke, seizures, or traumatic brain injury (which was defined as a head injury that results in unconsciousness lasting longer than 5 min or necessitates medical attention).
- Medication schedules that were not stable or involved usage of sedatives (such as opioids or benzodiazepines) and/or stimulants (such as amphetamine salts or methylphenidate) in the 5 days prior to the study visit.
- Current illicit substance use, schizoaffective disorder, bipolar disorder, history of alcoholism or frequent alcohol consumption (>14 drinks per week), as well as active suicidal thoughts etc.

### Neuropsychological tests

In addition to the LASSI-L, participants underwent a battery of neuropsychological tests traditionally used in HD research including the Stroop Word Reading Test (SWRT), Mini-Mental State Exam (MMSE), and Symbol Digit Modalities Test (SDMT).

#### LASSI-L methodology

The LASSI-L is a novel verbal learning paradigm designed to maximize the number of originally encoded items and elicit interference and intrusions in a time-restricted manner. This is achieved through semantic cueing, free recall, and cued recall in an alternating method. The test incorporates three semantic categories—fruits, clothes, and musical instruments—which are included in both of the test’s two sets of 15 words, known as List A and List B. The administration of the LASSI-L involves several distinct phases. Each item from List A is initially presented on a separate card, and the subject is asked to read each word aloud, engaging both visual and auditory recognition to facilitate active encoding. Following this presentation, the participant is asked to perform a free recall, attempting to remember all 15 terms within 60 s. Subsequently, the subject is asked to recall items from each of the three semantic categories one at a time, with a 20-s response restriction for each category. The total number of correctly recalled items is recorded for both free and cued recall following the initial presentation, referred to as A1-Free Recall and A1-Cued Recall, respectively. The same list (List A) is then presented again, and the subject undergoes the same procedure, with the results listed under A2. After this, a completely different set of 15 words (List B) is introduced, following the same three categories (fruits, clothes, and musical instruments). This process is repeated to obtain measurements labeled B1 and B2. Following the completion of List B, the subject is asked to recall the first List A using both cued and free recall methods, referred to as A3, without any further presentation of List A. Finally, after a 20-min delay, the subject is asked to perform a free recall of any items from either list, known as the Delayed Recall.

The LASSI-L measures several types of interference and memory abilities. Proactive Semantic Interference (PSI) is assessed by calculating the interference of the learned List A on the initial recall capacity of List B, with PSI being equivalent to B1. The test also measures the Failure to Recover from PSI (*fr*PSI) by assessing memory abilities following the second presentation of List B, labeled as *fr*PSI = B2 cued. Retroactive Semantic Interference (RSI) is evaluated by measuring the interference of learned List B on the subsequent cued recall of List A, with RSI corresponding to A3 cued. The overall effects of both types of interference are captured in the final segment of the Delayed Free Recall, which consists of items from either List A or List B. Throughout the test, the number of correctly recalled words and intrusions are meticulously tracked for each recall portion.

### Statistical analysis

Group differences in demographics and standard cognitive tests (SWRT, SDMT, MMSE) were analyzed using one-way ANOVA for independent measures, while sex, as a categorical variable, was compared using the Chi-Square (χ^2^) test. Given that demographic comparisons showed significant age differences across groups, multiple one-way ANCOVAs were performed to assess performance across all LASSI-L recall subsections, with group as the independent variable and age as a covariate. Where significant, additional pairwise comparisons between groups were conducted using unpaired t-test and corrected using the Bonferroni method. Given that the intrusion sections were not normally distributed, scores were compared using the Chi-Square (χ^2^) test, followed by additional pairwise Chi-Square comparisons with Bonferroni correction for multiple comparisons. To further control for multiple comparisons across all 18 sections of the LASSI-L, an additional correction was applied to the results using Bonferroni. To categorize the HD cohort into predictive stages, we employed the PIN score as a staging tool (23). The PIN score was developed as a prognostic index for motor diagnosis in HD and has previously been used to stratify premanifest HD cohorts for selection into clinical trials (24). The PIN score was chosen for its integration of both motor and cognitive measures, with particular emphasis on the SDMT, aligning with this study’s focus on characterizing the progressive cognitive changes associated with HD pathology. Pre-motor manifest participants were separated into two groups: (1) > 10 years from manifest HD and (2) < 10 years from manifest HD. The PIN score equation used was the following: (51 × TMS − 34 × SDMT +7 × age × (CAG − 34) − 883) ÷ 1,044 (23). TMS was developed as one domain of the UHDRSⓇ and used extensively in HD-related research and trials as a clinical rating scale of motor performance in HD (25). No participants in this study met the criteria for a PIN score of 0. Participants who had a PIN score of <0 were labeled as “> 10 years” from diagnosis and participants with PIN >0 were labeled as “< 10 years” (23). Any participant with TMS ≥ 10 was automatically categorized as belonging to the Manifest HD group.

To examine the sequence of interference effects in HD at the individual level, we established a threshold of 1.5 standard deviations (SD) below the control group’s mean performance as the criterion for significant deficits in PSI, *fr*PSI, and RSI. We then evaluated whether each participant exceeded this 1.5 SD cutoff for each stage (i.e., PSI, *fr*PSI, and RSI). By analyzing the pattern of deficits across the HD cohort, we identified the proportion of participants who followed the sequential pattern of impairment: PSI →* fr*PSI → RSI.

## Results

**Figure 1:**
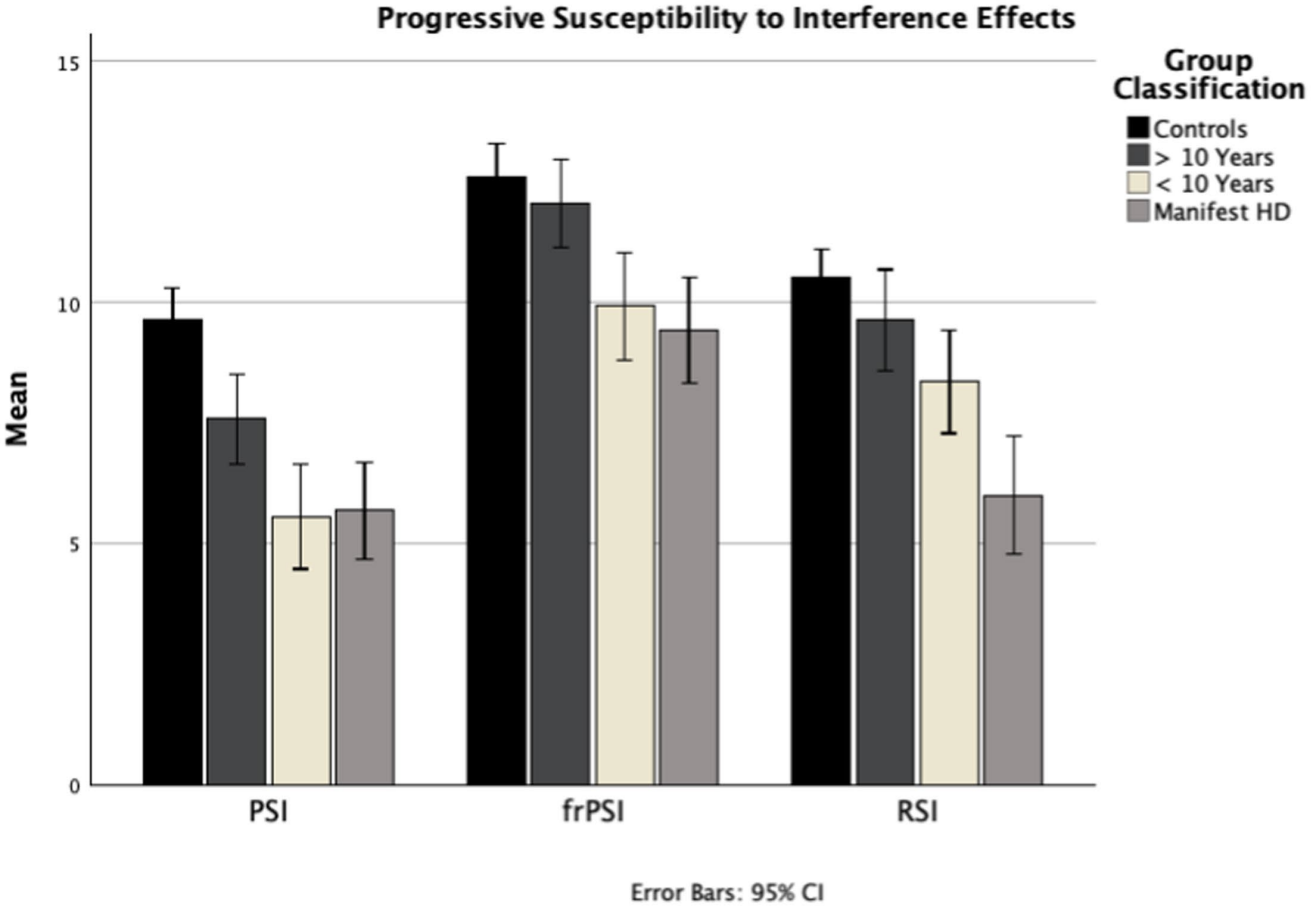
B_CR_1, Proactive Semantic Interference (PSI); B_CR_2, Failure to Recover from Proactive Semantic Interference (*fr*PSI); A_CR_3, Retroactive Semantic Interference (RSI); PIN, Prognostic index; PSI, Proactive Semantic Interference; *fr*PSI, Failure to Recover from PSI; RSI, Retroactive Semantic Interference.

A total of 151 participants (89 HD and 62 HC) were enrolled at BIDMC, UCSD, and the University of Miami. Using PIN staging and TMS, the HD cohort was subcategorized into >10 years from manifest HD (*n* = 38), <10 years from manifest HD (*n* = 23), and Manifest HD (*n* = 28). Demographic and standard cognitive test results per subgroup can be seen in Table 1. As expected given disease staging groups, Age (*f =* 15.88, *p* < 0.001), PIN: (*f* = 141.41, *p* < 0.001), and TMS (*f* = 125.10, *p* < 0.001) were statistically significant between groups. All standard cognitive measures were statistically significant between groups, Stroop Word Reading Test (*f* = 17.42, *p* < 0.001), Mini Mental State Exam (*f =* 47.02, *p* < 0.001), Symbol Digit Modalities Test (*f =* 22.46, *p* < 0.001). Group differences on the LASSI-L and pairwise comparisons can be seen in Table 2 and Figure 1.

**Table 1:** Demographics and baseline cognitive performance.

	Mean (SD)				F/χ^2^ (*p*-value)
	>10 Years (*N* = 38)	<10 Years (*N* = 23)	Manifest-HD (*N* = 28)	Control (*N* = 62)	
Age	37.84 (11.05)	47.04 (10.57)	56.89 (9.64)	44.00 (40.40)	**15.88 (<0.001)**
Education	15.42 (2.41)	16.87 (2.83)	15.75 (2.22)	15.84 (3.26)	1.30 (0.28)
Sex (Female)	58%	65%	57%	58%	0.45 (0.93)
CAG	41.76 (2.05)	43.00 (2.63)	42.89 (3.01)	–	2.37 (0.10)
TMS	0.95 (1.54)	3.61 (2.79)	22.50 (9.74)	–	**125.10 (<0.001)**
PIN	−0.88 (0.59)	0.63 (0.50)	1.16 (0.22)	–	**141.41 (<0.001)**
SWRT	98.08 (15.85)	85.13 (23.99)	67.93 (23.82)	100.29 (18.50)	**17.42 (<0.001)**
MMSE	28.58 (1.24)	28.04 (1.82)	29.14 (1.20)	29.14 (1.20)	**47.02 (<0.001)**
SDMT	59.87 (9.89)	43.43 (9.07)	30.93 (10.48)	56.69 (12.77)	**22.46 (<0.001)**

CAG, Cytosine-Adenine-Guanine; TMS, Total Motor Score; PIN, Prognostic index; SWRT, Stroop Word Reading Test; MMSE, Mini-Mental State Exam; SDMT, Symbol Digit Modalities Test. Bold results indicate statistical significance.

**Table 2:** LASSI-L performance comparison across stages of HD.

		Mean (SD)			F/χ^2^ (*p*-value)
	Control (*N* = 62)	>10 Years (*N* = 38)	<10 Years (*N* = 23)	Manifest-HD (*N* = 28)	
List A FR 1	9.97 (2.66)	9.71 (2.70)	8.04 (2.97)	7.79 (2.83)	**4.99 (<0.001)**
List A FR Int	0.31 (0.59)	0.08 (0.27)	0.43 (0.66)	0.39 (0.63)	12.20 (0.20)
List A CR 1	11.52 (2.48)	10.84 (2.68)	8.87 (2.72)	8.93 (3.02)	**7.19 (<0.001)**
List A CR 1 Int	0.47 (0.50)	0.26 (0.45)	0.61 (0.50)	0.39 (0.50)	7.84 (0.05)
List A CR 2	14.13 (1.29)	13.61 (1.97)	12.87 (1.89)	11.46 (2.84)	**9.76 (<0.001)**
List A CR 2 Int	0.16 (0.37)	0.08 (0.27)	0.52 (0.51)	0.29 (0.46)	**18.65 (<0.001)**
List B FR 1	8.61 (2.58)	7.24 (2.42)	5.70 (2.23)	5.25 (2.14)	**13.66 (<0.001)**
List B Fr 1 Int	0.37 (0.49)	0.32 (0.47)	0.43 (0.51)	0.71 (0.46)	**12.14 (0.007)**
List B CR 1 (PSI)	9.59 (0.33)	7.58 (2.82)	5.57 (2.52)	5.68 (2.60)	**17.42 (<0.001)**
List B CR 1 Int	0.65 (0.48)	0.55 (0.50)	0.83 (0.39)	0.86 (0.36)	**9.56 (0.02)**
List B CR 2 (*fr*PSI)	12.58 (2.79)	12.05 (2.76)	9.91 (2.56)	9.43 (2.82)	**8.83 (<0.001)**
List B CR 2 Int	0.55 (0.50)	0.61 (0.50)	0.78 (0.42)	0.68 (0.48)	4.38 (0.22)
List A FR 3	8.42 (3.31)	8.05 (3.47)	5.57 (2.86)	4.50 (2.70)	**10.60 (<0.001)**
List A FR 3 Int	0.53 (0.50)	0.47 (0.51)	0.78 (0.42)	0.57 (0.50)	5.99 (0.11)
List A CR 3 (RSI)	10.50 (0.34)	9.63 (3.18)	8.35 (2.46)	6.00 (3.16)	**15.15 (<0.001)**
List A CR 3 Int	0.74 (0.44)	0.58 (0.50)	0.74 (0.45)	0.82 (0.39)	5.30 (0.15)
Delayed FR	22.85 (4.46)	20.39 (5.30)	17.96 (4.49)	14.71 (5.83)	**14.11 (<0.001)**
Delayed FR Int	0.29 (0.46)	0.16 (0.37)	0.43 (0.51)	0.32 (0.48)	5.74 (0.13)

FR, Free Recall; CR, Cued Recall; PSI, Proactive Semantic Interference; *fr*PSI, Failure to Recover from PSI; RSI, Retroactive Semantic Interference; Int, Intrusion. Bold results indicate statistical significance.

Multiple ANCOVAs with age as a covariate, followed by post-hoc pairwise comparisons between groups, revealed significant deficits in PSI across all HD groups compared to controls. In the <10 years from manifest HD group, several additional sections also showed significant differences from controls, including List A Cued Recall 1, List A Cued Recall Intrusions, List B Free Recall, *fr*PSI, and Delayed Recall. In the Manifest HD group, participants struggled with most sections of the test, with deficits appearing in additional sections such as List A Cued Recall 2, List A Free Recall 3, and RSI (Table 3). With regard to interference patterns, the vast majority (98%, 87/89) of HD participants exhibited significant deficits in proactive interference (PSI) first. In other words, nearly every HD participant who showed deficits in *fr*PSI or RSI had also already demonstrated significant deficits in PSI. Those who did not meet the 1.5 SD threshold for PSI generally did not exhibit significant deficits in the other interference sections. Additionally, the majority (88%, 78/89) of HD participants demonstrated the following sequential pattern of susceptibility to interference: proactive interference (PSI) first, failure to recover from PSI (*fr*PSI) second, and retroactive interference (RSI) third.

**Table 3:** Pairwise group comparison of HD vs. controls at 3 disease stages.

***p*-value (Bonferroni corrected)**			
	**>10 Years** **(*N* = 38)**	**<10 Years** **(*N* = 23)**	**Manifest-HD** **(*N* = 28)**
List A FR 1	>	0.04 (>0.05)	0.05 (>0.05)
List A CR 1	>	***p* < 0.001 (*p* < 0.01)**	0.003 (>0.05)
List A CR 2	>	0.05 (>0.05)	***p* < 0.001 (*p* < 0.01)**
List A CR 2 Int	>	**0.002 (0.04)**	>
List B FR 1	0.009 (> 0.05)	***p* < 0.001 (*p* < 0.01)**	***p* < 0.001 (*p* < 0.01)**
List B FR Int	>	>	>
List B CR 1 (PSI)	***p* < 0.001 (*p* < 0.01)**	***p* < 0.001 (*p* < 0.01)**	***p* < 0.001 (*p* < 0.01)**
List B CR 1 Int	>	>	>
List B CR 2 (*fr*PSI)	>	***p* < 0.001 (*p* < 0.01)**	***p* < 0.001 (*p* < 0.01)**
List A FR 3	>	0.01 (>0.05)	***p* < 0.001 (*p* < 0.01)**
List A CR 3 (RSI)	>	0.01 (>0.05)	***p* < 0.001 (*p* < 0.01)**
Delayed FR	>	***p* < 0.001 (*p* < 0.01)**	***p* < 0.001 (*p* < 0.01)**
**Cognitive test comparisons**			
SWRT	>	0.02 (>0.05)	***p* < 0.001 (*p* < 0.01)**
MMSE	>	>	***p* < 0.001 (*p* < 0.01)**
SDMT	>	***p* < 0.001 (*p* < 0.01)**	***p* < 0.001 (*p* < 0.01)**

>, >0.05 prior to multiple comparison; FR, Free Recall; CR, Cued Recall; PSI, Proactive Semantic Interference; *fr*PSI, Failure to Recover from PSI; RSI, Retroactive Semantic Interference; PIE, Percentage of Intrusion Errors; Int, Intrusion; SWRT, Stroop Word Reading Test; MMSE, Mini-Mental State Exam; SDMT, Symbol Digit Modalities Test. Bold results indicate statistical significance.

## Discussion

In this study, the LASSI-L revealed a consistent pattern of escalating deficits across multiple aspects of verbal memory, including a sequential worsening of interference effects, as HD progresses from presymptomatic to manifest stages (Figure 1). The progression of susceptibility to verbal memory semantic interference in HD reliably follows three sequential stages: Proactive Semantic Interference (PSI), Failure to Recover from Proactive Semantic Interference (*fr*PSI), and finally Retroactive Semantic Interference (RSI). At >10 years from manifest HD (mean PIN −0.88; mean TMS 0.95), participants already exhibit significant susceptibility for proactive interference, consistent with prior studies (4). At <10 years from HD (mean PIN 0.63; mean TMS 3.61), deficits in *fr*PSI begin to accrue, indicating that these participants are susceptible to the persistent interference effect of List A despite two presentations of List B. By the symptomatic manifest stage (average TMS 22.50), in addition to the above deficits, participants also exhibit significant susceptibility to retroactive semantic interference (RSI). They demonstrate increased difficulty retrieving previously-encoded words from List A, likely due to a combination of poor encoding of List A and additional interference from List B. Notably, 98% (87/89) of participants with HD exhibited either normal performance, significant deficits in PSI alone, or deficits in PSI and either RSI or *fr*PSI, pointing to a sequential pattern with susceptibility to proactive semantic interference emerging as the first sign of cognitive decline. A large majority of HD participants (88%) adhered to the overall sequential interference pattern outlined above (PSI → *fr*PSI → RSI).

### Incremental interference deficits

Consistent with prior studies, PSI emerges as the earliest LASSI-L domain to exhibit a significant decline in HD, manifesting well before motor symptoms in the premanifest stage (4, 21). PSI on the LASSI-L comprises three important elements that may help account for this result:

1. **Active Encoding**: Involves presenting word lists twice in multiple domains (audio and visual) using explicit semantic categories given during instructions, which maximizes the initial encoding of List A, thereby increasing the potential for subsequent proactive interference (26).
2. **Proactive Interference**: Defined as the inability to suppress competing or irrelevant items, where previously acquired knowledge obstructs the acquisition of new information (27–29).
3. **Salient Semantic Relatedness**: Refers to the strong and intuitive connections between categories of words that are easily accessible to participants. These inherent associations make it challenging for individuals to disengage from proactive interference, thereby intensifying the interference effect (19, 20, 30, 31).

Although PSI is the first section to challenge HD participants, participants in the >10 years from HD cohort do not exhibit significant difficulty with the initial retrieval of List A (A1), suggesting that their encoding ability remains relatively intact. However, their increased susceptibility to PSI likely stems from an early weakness with flexible updating of information and/or reduced working memory. Even at this early stage, the ability to efficiently manage multiple competing sets of information seems to be somewhat compromised. However, despite the proactive interference effect, HD participants demonstrated adequate inhibitory control, committing very few intrusion errors, such as recalling items from List A or naming other items within the same semantic category, with rates comparable to those of the control group. Furthermore, participants in this group perform similarly to controls when returning to List A a third time (A3) after encoding List B twice, suggesting that they are not yet susceptible to retroactive interference at this stage.

As participants near the motor-manifest stage (<10 years of HD onset), the inability to recover from proactive interference (*fr*PSI)becomes increasingly evident. This persistent difficulty, even after a second attempt to encode List B, could result from an increased or prolonged susceptibility to proactive semantic interference, coupled with a diminished ability to recruit the cognitive control resources that were effective in earlier stages (32). This pattern is also observed in individuals with amnestic mild cognitive impairment (MCI) who are at risk for Alzheimer’s disease (32). However, unlike in MCI/AD, HD participants at this stage continue to exhibit very few intrusion errors, suggesting that source memory encoding and inhibitory mechanisms are less affected. In other words, while HD participants may struggle with less efficient encoding and a reduced capacity to update working memory, they do not appear to experience increased confusion regarding the source of these memories.

By the motor manifest stage, participants also exhibit significant difficulties with RSI, where they fail to retrieve previously encoded words (i.e., List A) due to disruption from newly acquired information. RSI measures susceptibility to retroactive interference (28, 29), where participants likely suppress previously learned words (List A) after being exposed to a new list (List B) followed by a short delay. Similar to PSI, susceptibility to RSI on the LASSI-L is enhanced by the use of semantically-related word lists (32). Notably, despite a significant decline in performance across most sections of the LASSI-L, the manifest HD group remains relatively resistant to committing intrusion errors. The only section where they tended to make more intrusion errors was, perhaps unsurprisingly, during List B1 Free Recall after switching to List B; however, even this did not reach statistical significance after correction for multiple comparisons. This pattern suggests a relatively preserved ability to bind encoded items to the correct word list, even at more advanced stages of the disease.

### Additional progressive deficits on LASSI-L

Beyond the susceptibility to interference effects captured by PSI, *fr*PSI, and RSI, the <10 years from HD group exhibited deficits in List A Cued Recall 1, List A Cued Recall 2 Intrusions, List B Free Recall, and Delayed Recall. The decline in List B Free Recall performance highlights a progressively increasing susceptibility to proactive interference, as performance deteriorates even in the absence of semantic cueing. Difficulties with Delayed Recall, which involves retrieving both lists, are expected given the earlier difficulties with encoding List B; if participants struggled to encode List B, it follows that they would also struggle to retrieve it after a delay. Unlike most other sections that show a steady decline in performance over time, List A Cued Recall 2 Intrusions—initially significantly different from controls—are no longer significant in the manifest HD group. The reason for this is unclear but could be a transient finding, potentially due to individuals at this stage of HD responding more quickly than those in the manifest stage, resulting in better performance but also a higher likelihood of errors. However, this warrants further investigation in larger samples before drawing firm conclusions. In the Manifest HD group, additional difficulties were observed. Deficits in List A Cued Recall 2 suggest the onset of significant encoding impairments despite multiple trials. Similarly, deficits in List A Free Recall 3, like those recorded in RSI, indicate an increased susceptibility to retroactive interference, which, at this stage of the disease, occurs even without the need for semantic cueing.

The progression of verbal memory deficits in Huntington’s disease (HD) aligns with the well-established pattern of sequential brain atrophy, already integrated into existing staging systems. While not directly analyzed in our study, early atrophy in regions like the caudate, putamen, and globus pallidus likely disrupts fronto-striatal circuits essential for executive function (33). This disruption may manifest as difficulty suppressing irrelevant information during verbal memory tasks (34), increasing susceptibility to proactive semantic interference (PSI). At early stages, limited atrophy allows recruitment of unaffected cognitive control circuits, enabling individuals to overcome interference. As HD progresses and atrophy spreads to subcortical regions like the substantia nigra (33), cognitive flexibility and adaptability decline. Degeneration of the substantia nigra, key for learning and adaptability, may result in persistent interference (*fr*PSI). By the manifest stage, widespread atrophy, including in the hippocampus (33), likely leads to less efficient encoding, heightening vulnerability to retroactive semantic interference (RSI).

### Limitations

While our study highlights the progression of memory deficits in HD, several limitations should be considered. Although the LASSI-L effectively targets semantic interference, it may not capture all aspects of memory dysfunction in HD. The absence of forced-choice or yes/no recognition tasks also limits our ability to differentiate between encoding and retrieval deficits. Additionally, while participants spanned all stages of HD, the lack of longitudinal data prevents verification of the sequential pattern of deficits (PSI → *fr*PSI → RSI) at the individual level.

Furthermore, the study did not explicitly measure processing speed, a common issue in HD, leaving uncertainty about how varying response times might have affected results. Without brain imaging, we also lack insight into how these cognitive findings relate to existing imaging-based staging systems.

Future research could address these limitations by adjusting the LASSI-L methodology, such as incorporating variations without semantic cues or allowing extended response times. A longitudinal study tracking LASSI-L performance over time would also clarify the sequential progression of deficits.

## Conclusion

The LASSI-L reveals a consistent, sequential pattern of susceptibility to interference and verbal memory deficits in Huntington’s disease (HD). Individuals more than 10 years from predicted onset exhibit proactive interference but can still compensate, highlighting early cognitive changes. As they move to within 10 years of onset, overcoming proactive semantic interference (*fr*PSI) becomes more challenging, and by the manifest stage, retroactive semantic interference (RSI) and broader verbal memory deficits emerge. Notably, even at the manifest stage, HD participants show relative resistance to intrusion errors, contrasting with findings in MCI/AD studies (30).

The LASSI-L’s design—featuring brief, timed sections, semantic categories, and an emphasis on cueing and active encoding—likely underpins the observed memory deficit patterns, which may be overlooked by traditional assessments. These findings suggest that the LASSI-L could enhance staging systems like the HD-ISS by reliably capturing cognitive decline at multiple stages of disease progression.

## Data Availability

All data supporting the conclusions of this article may be requested from the corresponding author.
